# Impact of Branched-Chain Amino Acid Catabolism on Fatty Acid and Alkene Biosynthesis in *Micrococcus luteus*

**DOI:** 10.3389/fmicb.2018.00374

**Published:** 2018-03-12

**Authors:** Maximilian J. Surger, Angel Angelov, Philipp Stier, Maria Übelacker, Wolfgang Liebl

**Affiliations:** Department of Microbiology, Technical University of Munich, Munich, Germany

**Keywords:** *Micrococcus luteus*, branched fatty acid synthesis, olefins, isovaleryl-CoA, isobutyryl-CoA, 2-methylbutyryl-CoA, BCKD complex, branched amino acid catabolism

## Abstract

*Micrococcus luteus* naturally produces alkenes, unsaturated aliphatic hydrocarbons, and represents a promising host to produce hydrocarbons as constituents of biofuels and lubricants. In this work, we identify the genes for key enzymes of the branched-chain amino acid catabolism in *M. luteus*, whose first metabolic steps lead also to the formation of primer molecules for branched-chain fatty acid and olefin biosynthesis, and demonstrate how these genes can be used to manipulate the production of specific olefins in this organism. We constructed mutants of several gene candidates involved in the branched-chain amino acid metabolism or its regulation and investigated the resulting changes in the cellular fatty acid and olefin profiles by GC/MS. The gene cluster encoding the components of the branched-chain α-keto acid dehydrogenase (BCKD) complex was identified by deletion and promoter exchange mutagenesis. Overexpression of the BCKD gene cluster resulted in about threefold increased olefin production whereas deletion of the cluster led to a drastic reduction in branched-chain fatty acid content and a complete loss of olefin production. The specificities of the acyl-CoA dehydrogenases of the branched amino acid degradation pathways were deduced from the fatty acid and olefin profiles of the respective deletion mutant strains. In addition, growth experiments with branched amino acids as the only nitrogen source were carried out with the mutants in order to confirm our annotations. Both the deletion mutant of the BCKD complex, responsible for the further degradation of all three branched-chain amino acids, as well as the deletion mutant of the proposed isovaleryl-CoA dehydrogenase (specific for leucine degradation) were not able to grow on leucine in contrast to the parental strain. In conclusion, our experiments allow the unambigous assignment of specific functions to the genes for key enzymes of the branched-chain amino acid metabolism of *M. luteus*. We also show how this knowledge can be used to engineer the isomeric composition and the chain lengths of the olefins produced by this organism.

## Introduction

Aliphatic hydrocarbons are attractive target compounds for microbial production and its improvement by metabolic engineering. They represent an interesting alternative to plant oils as source for biofuels and other oleochemicals. Microbial hydrocarbon and oil production is sustainable and can be carried out without competition with food production. The obtained molecules can be very similar or identical to the main components of petroleum-derived fuels and this makes them fully compatible with the existing storage and distribution infrastructure (Pfleger et al., 2015).

Although hydrocarbon biosynthesis in bacteria has been known for decades (Tornabene et al., 1967; Albro and Dittmer, 1969), elucidation of the underlying biochemical mechanisms has only begun recently. One of the routes, described initially in “*Sarcina lutea*” (now *Kocuria rhizophila*), is encoded by a gene cluster designated as *oleABCD* which was first identified in *M. luteus* NCTC2665 (Beller et al., 2010). Orthologous gene clusters were later found in various other bacteria (Sukovich et al., 2010). According to the current state of knowledge, two fatty acyl-CoA molecules serve as substrates for a non-decarboxylative head-to-head Claisen condensation to a β-ketoacid, catalyzed by a condensing thiolase (OleA). This β-keto-acid is reduced by OleD, a NADH-dependent dehydrogenase. The resulting β-hydroxy-acid is converted to a β-lactone by OleC, a β-lactone synthase, and finally decarboxylated to an internal olefin by OleB, an AMP-dependent synthase/ligase. The double bond is located at the connection point of the two fatty acid precursors in the center of the olefin molecule (Christenson et al., 2016).

Because fatty acyl chain length, degree of saturation and branching are key to the properties of the derived microbial hydrocarbons, the understanding of the mechanisms that modulate the type of fatty acids is critical for the ability to design hydrocarbons with defined structures. The olefins produced by *M. luteus* are terminally (*iso*- or *anteiso*-) methyl-branched, and it can be presumed that the type of branching depends primarily on the availability of short- and branched-chain acyl-CoA primers for fatty acid biosynthesis. Structural work on the β-ketoacyl-ACP synthase III (FabH) from *M. luteus*, which catalyzes the first step of fatty acid elongation, indicates that this enzyme favors the binding of branched rather than straight chain (*sc*) acyl-CoA primers (Pereira et al., 2012). These acyl-CoA primers are derived from branched amino acids (BCAA) and represent intermediates of the BCAA degradation. The BCAA are first converted to α-keto acids by a branched-chain amino acid transaminase, followed by decarboxylation by the branched-chain α-keto acid dehydrogenase complex (BCKD), resulting in branched-chain acyl-CoA primers. In Gram-positive bacteria, these primers are alternatives to the straight-chain acetyl- and propionyl-CoA in the first elongation step of the fatty acid biosynthesis, the reaction of the β-ketoacyl-ACP synthase III (FabH). FabH from *M. luteus*, in contrast to Gram-negative bacteria, can process straight-chain as well as branched-chain acyl-CoA primers (Pereira et al., 2012). The relevance of the BCKD complex and of β-ketoacyl-ACP synthase III for branched-chain fatty acid synthesis was pointed out by Howard et al., who transferred the ability for branched-chain fatty acid synthesis to the Gram-negative bacterium *Escherichia coli* only by introducing the *Bacillus subtilis* genes for those two enzymes (Howard et al., 2013).

Since the 1970s, the degradation pathways of isoleucine, leucine and valine have been thoroughly studied in the low-GC (phylum *Firmicutes*) model bacterium *Bacillus subtilis*. In *B. subtilis*, the key enzymes that determine the fate of acyl-CoA primers in fatty acid biosynthesis have been identified (Willecke and Pardee, 1971; Massey et al., 1976; Oku and Kaneda, 1988; Kaneda, 1991).

In this report, we investigate the genes for key reactions of the BCAA catabolism in the high-GC Gram-positive bacterium (phylum *Actinobacteria*) *M. luteus*. To this end, it was of interest to identify in *M. luteus* the genes encoding the branched-chain alpha-keto acid dehydrogenase (BCKD) complex, which is a major player of the branched-chain fatty acid synthesizing system. We also sought to investigate the presence of an acyl-CoA synthase for activation of free branched-chain carboxylic acids, as an alternative pathway for branched fatty acid primer generation in addition to the BCKD-dependent amino acid decarboxylation route in *M. luteus*. To better understand the metabolism of the short branched acyl-CoA derivatives it was also of interest to identify the genes for the short branched-chain acyl-CoA dehydrogenases, which are involved in the degradation of the acyl-CoA intermediates formed from isoleucine, leucine or valine and thus compete for the same intermediates also used as primer molecules in fatty acid and olefin biosynthesis.

## Materials and Methods

### Bacterial Strains and Growth Conditions

A tryptophan auxotroph of the strain “*Micrococcus lysodeikticus*” (*M. luteus*) ISU, *trpE16* (Kloos and Schultes, 1969; Angelov et al., 2015), was grown in LB or modified Naylor medium at 30°C and used as host for DNA manipulations and also as a reference strain. The Naylor medium had a pH of 7.2 and contained per liter 10 g glutamate x H_2_O, 7 g glucose, 5 g NH_4_Cl, 2 g K_2_HPO_4_, 100 mg MgSO_4_ × 7H_2_O, 100 mg tryptophan, 10 mg biotin, 4 mg FeSO_4_ × 7H_2_O, 2 mg MnCl_2_ × 4H_2_O and was prepared with ultrapure water. For the growth studies with 20 mM amino acids as the only nitrogen source, NH_4_Cl and biotin were omitted. The *E. coli* strain XL1-Blue (Stratagene, La Jolla, CA, United States) was grown in LB medium at 37°C and was used for cloning purposes and propagation of recombinant plasmids. Where appropriate, the growth media were supplemented with kanamycin (60 μg/ml for *M. luteus*, 20 μg/ml for *E. coli*), hygromycin (100 μg/ml for *M. luteus*) and 5-fluorocytosine (500 μg/ml for *M. luteus*, dissolved in water).

### Bioinformatics Methods

Potential orthologs of branched-chain alpha-keto acid dehydrogenase (BCKD-) complex components, orthologs of BCAA degradation enzymes, and orthologs of short and branched-chain acyl-CoA dehydrogenases were predicted in the genome of *M. luteus* by blastp using the *nr* database (Altschul et al., 1997). Amino acid sequence alignments of short- and branched-chain acyl-CoA dehydrogenases were generated by Clustal Omega (Goujon et al., 2010) with a blosum62 matrix and Geneious (version 10.0.2) (Kearse et al., 2012).

### Construction of *M. luteus* Deletion and Promoter-Exchange Mutants

Homologous DNA sequence regions upstream and downstream of promoter-insertion sites as well as genes to be deleted, selection markers, and promoter sequences were amplified by PCR, using Q5 polymerase (New England Biolabs). The assembly of different PCR products with overlaps was achieved by Gibson Assembly (New England Biolabs). The *in vitro* Gibson assembly reactions (∼0.2–0.4 μg DNA) were added directly to *M. luteus* cells for uptake *via* natural transformation and plated on LB plates supplemented with the appropriate antibiotic, as described before (Angelov et al., 2015). For construction of markerless deletion mutants, the *codAB* counterselection method of Kostner et al. (2013) was used. In this approach, amplified flanking regions of the gene to be deleted were fused together and cloned in the plasmid pKOS6b using Gibson Assembly. Chemically competent *E. coli* XL-1 cells were transformed with the Gibson Assembly reactions. The purified recombinant plasmid, carrying the deletion allele was transformed in naturally competent *M. luteus* cells and plasmid integrants were selected on LB plates supplemented with kanamycin. Counterselection was performed on LB plates containing 5-fluorocytosine and the correctness of the alterations in the genome of *M. luteus* were confirmed by PCR and sequencing of the target genomic regions (Angelov et al., 2013). The *M. luteus* strains used in this study are listed in **Table 1**.

**Table 1:** *M. luteus* strains used in this study.

Strain	Genotype and relevant phenotype	Source
trpE16	trpE16, Trp^-^ mutant of ATCC 27141	Kloos and Schultes, 1969
P_up_06800:kan	trpE16 with insertion of stronger Mlut_05030 promoter upstream of Mlut_06800; Kan^R^	This study
P_up_13320:kan	trpE16 with insertion of stronger Mlut_05030 promoter upstream of Mlut_13320; Kan^R^	This study
P_up_17810:kan	trpE16 with insertion of stronger succinate dehydrogenase promoter upstream of Mlut_17810; Kan^R^	This study
Δ06800-20:hyg	trpE16 with deletion of Mlut_06800-06820; Hyg^R^	This study
Δ06840	trpE16 with deletion of Mlut_06840	This study
Δ02820:kan	trpE16 with deletion of Mlut_02820; Kan^R^	This study
Δ02900:kan	trpE16 with deletion of Mlut_02900; Kan^R^	This study
Δ06870:kan	trpE16 with deletion of Mlut_06870; Kan*^R^*	This study
ope	trpE16 P*_up_*13230 (replacement of native *oleABCD* gene cluster promoter by strong succinate dehydrogenase promoter)	This study
ope Δ02880:hyg	ope with deletion of Mlut_02880; Hyg^R^	This study
ope Δ02900:kan	ope with deletion of Mlut_02900; Kan^R^	This study
ope Δ02880:hyg Δ02820:kan	ope with deletion of Mlut_02880 and Mlut_02820; Hyg^R^ Kan^R^	This study
ope Δ02880:hyg Δ02900:kan	ope with deletion of Mlut_02880 and Mlut_02900; Hyg^R^ Kan^R^	This study


### Olefin Extraction

*Micrococcus luteus* cultures were grown at 30°C to stationary phase and 10 ml samples were taken. The samples were centrifuged in glass vials with PTFE screw-caps. The cell pellets were re-suspended in residual supernatant and 100 μl 100% acetic acid was added and thoroughly mixed. One ml of methanol and 4 ml of hexane, amended with 10 or 50 μg/ml triacontane (Tokyo Chemical Industry) as an internal standard were added. The whole mixture was shaken overnight at 640 rpm in KS 130B shaker. To facilitate phase separation, the samples were centrifuged and the upper hexane phase was used for analysis.

### Lipid and Free Fatty Acid Extraction

Cultures were grown to stationary phase and 2.5 ml samples were transferred to glass vials with PTFE screw-caps. The cell suspension was thoroughly mixed with 100 μl 100% acetic acid. Then 500 μl of a 100 μg/ml eicosanoic acid (Larodan AB) solution in ethanol as internal standard and 5 ml of methanol/chloroform (1:1) were added. The whole mixture was shaken overnight at 640 rpm and after centrifugation the lower chloroform phase was transferred to a new glass vial and evaporated completely. Lipid and free fatty acids were methylated by the addition of 500 μl 1.2 M HCl in methanol and incubation at 50°C with shaking overnight. The methylation reaction was quenched by adding 5 ml of 100 mg/ml NaHCO_3 (aq)_. Finally, the fatty acid methyl esters (FAMEs) were extracted with 1 ml of hexane and the hexane phase was transferred into a GC/MS vial for analysis.

### Gas Chromatography/Mass Spectrometry (GC/MS)

GC/MS analysis was performed using a Trace GC Ultra gas chromatograph in combination with a DSQ mass spectrometer (Thermo Scientific). The gas chromatograph was equipped with a PTV splitless injection system and a Rxi-5 ms (30 m × 0.25 mm i.d. × 0.25 μm) capillary column, and the MS was operated under ionization by electron impact at 70 eV and 200°C. The samples (1 μl) were injected in split mode (split 1:10). Helium flow was maintained at 1 ml/min. The temperature of the column was started at 40°C for 3 min, increased to 250°C at 15°C/min, and held for 19 min. Mass spectra were recorded at m/z 40-600 at a rate of 0.8741/s.

Quantification of olefins was done by direct comparison of their peak areas with the peak area of triacontane (C30 alkane) of known concentration, which had been added to the hexane with which the olefins were extracted. Quantification of FAMEs was done by direct comparison of their peak areas with the peak area of eicosanoic acid (C20:0) of known concentration, which had been added to the sample at the beginning of the extraction procedure.

### RNA Extraction and Processing for qPCR or RNA Sequencing

For RNA extraction, the “ZR Fungal/Bacterial RNA MiniPrep” Kit (Zymo Research) and the corresponding protocol was used, using lysozyme treatment (at 1 μg/ml for 10 min) of the cells instead of bead beating for cell lysis. RNA was converted to cDNA by the “iScript Select cDNA Synthesis” Kit (BIO-RAD Laboratories). qPCR was conducted with “SsoAdvanced Universal SYBR Green Supermix” (BIO-RAD Laboratories). The relative quantification of the transcripts was performed with the 2^-ΔΔ^*C*_T_ method (Livak and Schmittgen, 2001).

## Results

Two steps of the BCAA catabolism were in the focus of our investigations. The first one, part of the common degradation pathway of all three BCAAs, is the reaction of the branched-chain α-keto acid dehydrogenase (BCKD) complex. The second step, which is part of the individual degradation pathways of those amino acids, is catalyzed by short- and branched-chain-specific acyl-CoA dehydrogenases (Massey et al., 1976).

### Identification of the Main Components of the Branched-Chain α-keto Acid Dehydrogenase (BCKD) Complex and Their Effect on Olefin Composition

The BCKD complex, using thiamine diphosphate, NAD^+^ and CoA-SH as cofactors, converts 3-methyl-2-oxopentanoate, 4-methyl-2-oxopentanoate and 3-methyl-2-oxobutanoate to 2-methylbutyryl-CoA, isovaleryl-CoA and isobutyryl-CoA, respectively, thereby releasing CO_2_. This type of reaction is analogous to the activities of the pyruvate and 2-oxoglutarate dehydrogenase complexes, which show also amino acid sequence conservation. The enzyme complex consists of four subunits: two subunits of the branched-chain α-keto acid decarboxylase (E1), a lipoamide acyltransferase (E2), and a lipoamide dehydrogenase unit (E3). In the genome of *Micrococcus luteus* three gene clusters could be found which potentially code for the pyruvate dehydrogenase-, 2-oxoglutarate dehydrogenase- and BCKD complexes: Mlut_06800-06820, Mlut_13320-13340 and Mlut_17810-17790 (numbering in the order of transcription of the genes of the operons). The Mlut_06800-06820 and Mlut_17810-17790 operons encode E1 and E2 subunits, while the Mlut_13320-13340 operon possesses only genes for E2 and E3 subunits. It is known that different dehydrogenase complexes can share the same E3 monomer (Namba et al., 1969; Wang et al., 1993).

**FIGURE 1:**
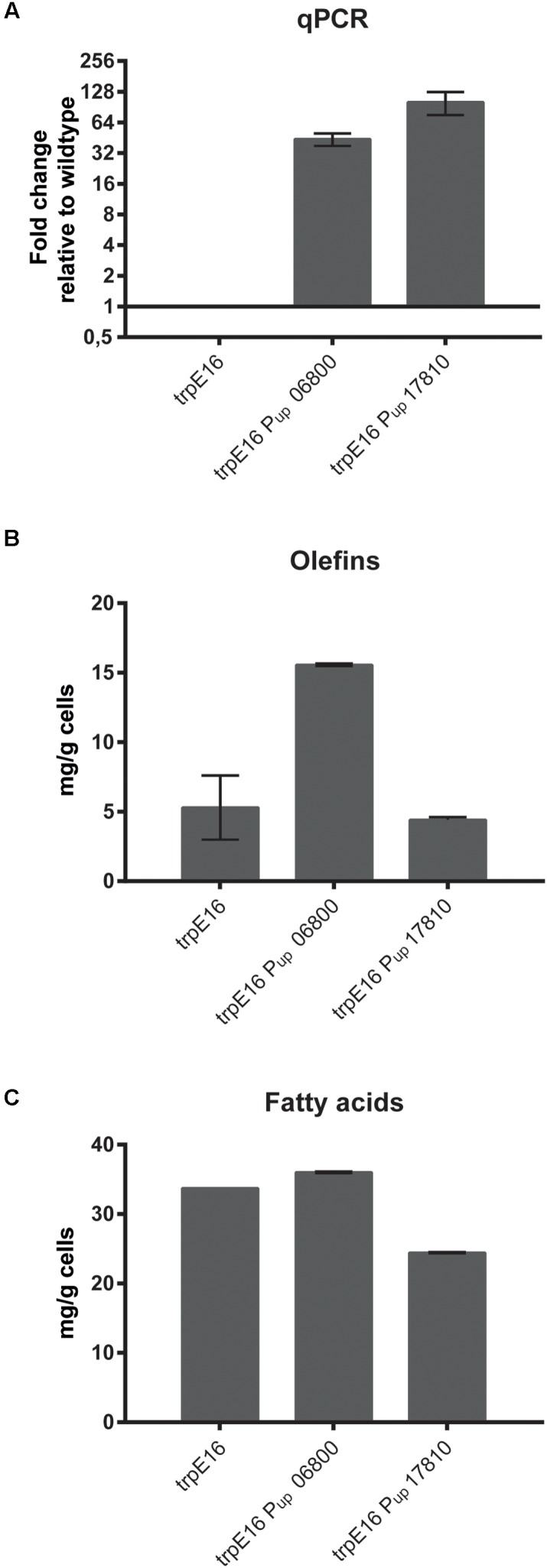
Promoter-up exchange mutants of *M. luteus* BCKD complex gene cluster candidates. Gene expression analysis by qPCR **(A)**, cellular olefin amounts **(B)** and cellular fatty acid amounts in minimal medium **(C)** after replacement of the native promoters with stronger promoters (see text) relative to the parental strain. The qPCR data is obtained from three technical measurements of two biological samples. In the case of the qPCR data a log_2_-scale was employed on the *y*-axis. The olefin and fatty acid concentrations were determined from two biological replicates. Error bars represent standard deviation.

In order to identify if one of the two candidate clusters with genes for the E1 and E2 subunits codes for the BCKD gene cluster we exchanged the promoter regions with potentially strong promoters (promoter region of Mlut_05030 in the case of Mlut_06800, promoter region of Mlut_04850 in the case of Mlut_17810; the promoter regions were chosen based on gene expression data derived from the microarray experiment of Pereira et al., 2012). Changes in the transcription level were checked by qPCR of the target genes after growth to exponential phase in minimal medium. Relative quantification by the 2^-ΔΔ*C*_T_^ method revealed that the genes Mlut_06800 and Mlut_17810 were strongly overexpressed in those mutants in comparison to the parental *trpE16* strain (**Figure 1A**). The GC/MS analysis of olefins produced by the promoter exchange mutants showed that overexpression of the gene cluster Mlut_06800-06820 led to a 3-fold increase of olefin production in our minimal medium. The promoter exchange mutations upstream of Mlut_17810 had no effect on olefin production (**Figure 1B**). Analysis of the fatty acids by GC/MS (**Figure 1C**) showed that the overexpression of cluster Mlut_06800-06820 had no effect on the fatty acid amount, while the promoter exchange mutation in Mlut_17810-17790 caused a drop of fatty acid production by about 30%. This is in line with the idea that an increased amount of active pyruvate- and 2-oxoglutarate-dehydrogenase would lead to induction of the citric acid cycle, which is in direct competition with the fatty acid synthesis for acetyl-CoA. The composition of the fatty acids as well as of the olefin isomers was not affected by the overexpression of Mlut_06800-06820 (data not shown).

**FIGURE 2:**
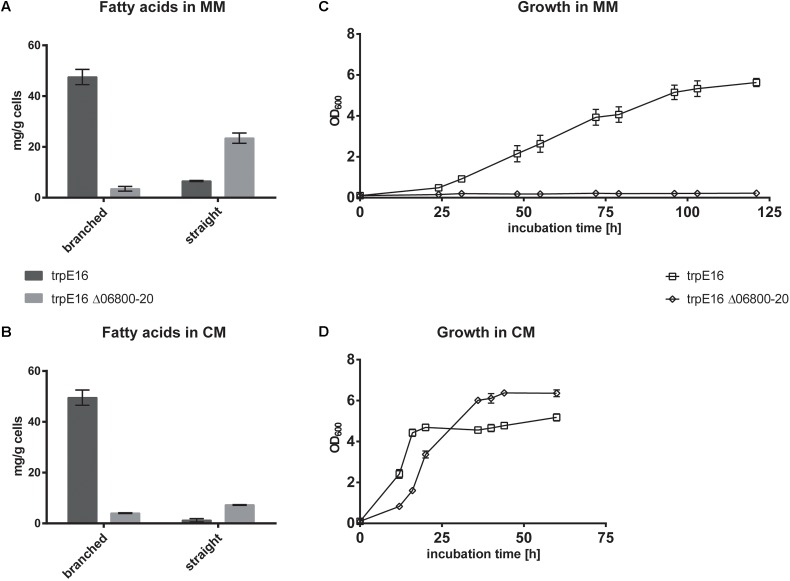
Branched and straight chain fatty acid amounts in wild type *M. luteus* trpE16 and in the BCKD deletion mutant trpE16 Δ06800-20:hyg grown in minimal medium (MM) **(A)** and complex medium (CM) **(B)**. Each bar represents the data from two biological replicates. Growth curves of trpE16 and the BCKD deletion mutant in minimal medium **(C)** and in complex medium **(D)**. Each curve represents data from three biological replicates, error bars represent standard deviation.

To further investigate the metabolic routes for branched-chain fatty acids synthesis in *M. luteus*, we generated a BCKD complex deletion strain in which the Mlut_06800-06820 gene cluster was replaced with a hygromycin resistance cassette. The deletion of the genes of the BCKD complex led to a strong growth inhibition of the strain in minimal medium, while in complex medium normal growth was observed. In both media, no olefins could be detected in this strain. In our standard minimal medium, a strong shift in the fatty acid composition was observed, with more than 80% of the fatty acids being straight chain, as opposed to the wild type where the fraction of *sc* fatty acids was less than 16%. This shift was accompanied by a significant decrease in the total amount of fatty acids (**Figure 2A**). Similar phenotype was also observed in complex medium, where the total amount of fatty acids produced in the BCKD mutant was drastically reduced to less than 20% of that of the parental strain and the fraction of the *sc* fatty acids was more than 60% (**Figure 2**). These dramatic changes in the fatty acid composition and the predominance of straight over branched fatty acids may have reached a critical level in minimal medium. The requirement of such a critical level of branched-chain fatty acids for growth has been demonstrated in *B. subtilis* (Kaneda, 1988). Branched-chain fatty acids in Gram-positive bacteria as well as unsaturated straight chain fatty acids in Gram-negative bacteria, which are incorporated into membrane lipids, contribute to an increased membrane fluidity due to their low phase transition temperatures (Kaneda, 1991). It seems that in the case of the BCKD mutant in minimal medium the fraction of branched-chain fatty acids in the membrane lipids has been reduced to such an extent that cell division is inhibited, probably due to the reduced membrane fluidity.

**FIGURE 3:**
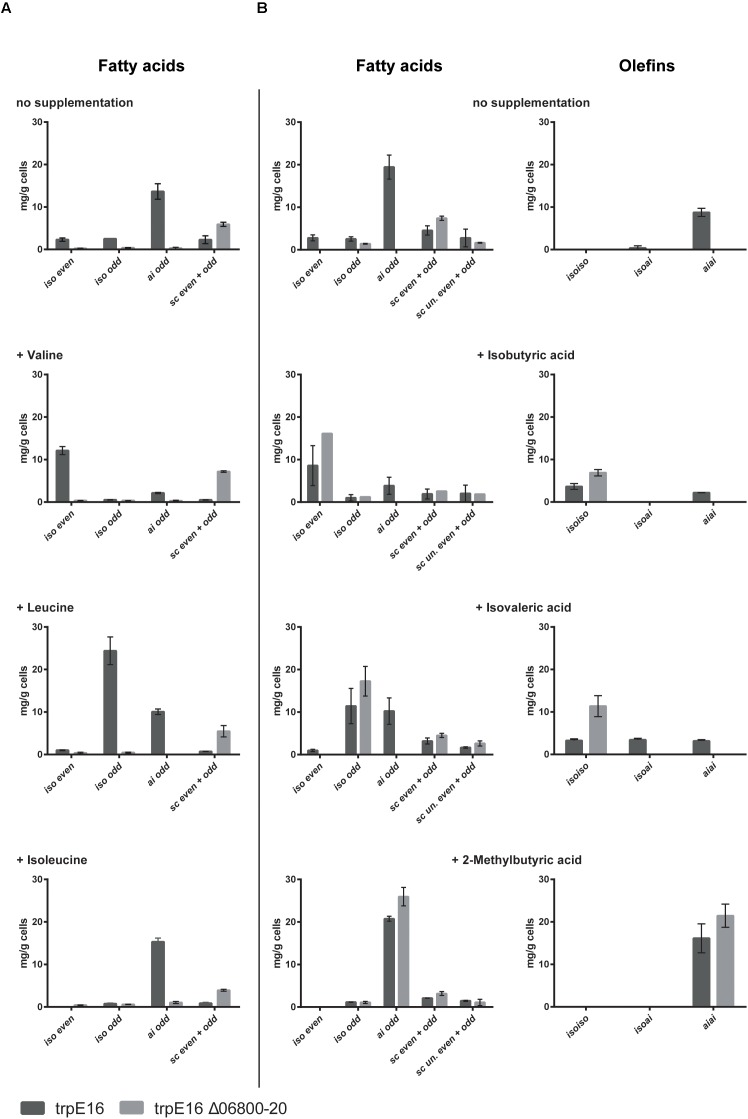
Fatty acid and olefin amounts in the BCKD deletion mutant *M. luteus* trpE16 Δ06800-20:hyg compared to the parental strain *M. luteus* trpE16 grown in minimal medium. **(A)** Effects of supplementation with branched-chain amino acids. **(B)** Effects of supplementation with short branched-chain carboxylic acids. Both supplementations were performed with 4 mM of each compound. Each bar represents the data from two biological replicates, error bars represent standard deviation. Abbreviations: *iso* even, *iso*-branched even-numbered; *iso* odd, *iso*-branched odd-numbered; *ai* odd, *ai*-branched odd-numbered; *sc* even + odd, straight-chain even- and odd-numbered; *sc* un. even + odd, straight-chain unsaturated even- and odd-numbered.

Supplementation of the minimal medium with valine, leucine or isoleucine (at 4 mM) led to increased production of the corresponding branched fatty acids in the parental strain *trpE16* but not in the BCKD deletion mutant (**Figure 3A**). This result further supports our assignment of the Mlut_06800-06820 genes as encoding the components of the BCKD complex. The observed low level of branched fatty acids in the BCKD deletion mutant in minimal medium could result from unspecific activity of the pyruvate- or the 2-oxoglutarate dehydrogenase complexes.

An alternative route to produce branched fatty acid primer molecules are short- and branched-chain specific acyl-CoA synthetases, which activate externally supplied short branched-chain carboxylic acids directly to the corresponding fatty acid primer molecules. To test if this alternative route is present in *M. luteus* trpE16 and is still active in the ΔBCKD mutant, the mutant as well as the parental strain were supplemented with 2-methylpropanoic (isobutyric) acid, 3-methylbutyric (isovaleric) acid or 2-methylbutyric acid (at 4 mM) in minimal medium. In both strains the supplementation allowed the synthesis of the corresponding branched fatty acids, and in the case of the deletion mutant the effect of the supplementation was more pronounced because of the missing background production (**Figure 3B**). In addition, these supplementations restored the olefin production defect in the mutant, leading to olefin isoforms corresponding to the supplied precursor (**Figure 3B**).

### Identification of Short- and Branched-Chain-Specific acyl-CoA Dehydrogenases and Their Influence on Fatty Acids and Olefin Composition

The branched fatty acid primer molecules 2-methylbutyryl-CoA, 3-methylbutyryl-CoA (isovaleryl-CoA) and 2-methylpropanoyl-CoA (isobutyryl-CoA) are also intermediates in the degradation pathway of the BCAAs isoleucine, leucine and valine, respectively. In the supplementation experiment above we observed that the availability of these primers can have a major impact on the isomeric composition of the olefins produced by *M. luteus*. In *B. subtilis*, BCAAs degradation follows a common pathway up to the level of the activated acyl-CoA derivatives which are further catabolized by specific acyl-CoA dehydrogenases (Massey et al., 1976). We reasoned that the deletion of specific dehydrogenases of the BCAA degradation pathways could lead to an increased supply of the respective acyl-CoA for fatty acid and olefin biosynthesis and could be used as a means to engineer the branching pattern of the olefins of *Micrococcus*.

**FIGURE 4:**
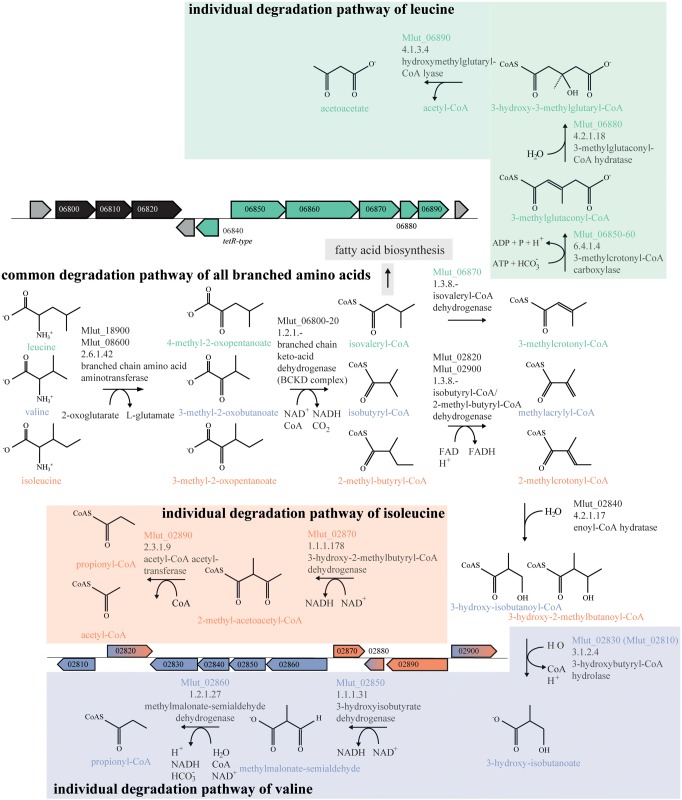
Branched-chain amino acid (BCAA) degradation pathways in *M. luteus.* Metabolites genes and gene clusters written or dyed in green are part of the leucine degradation pathway, those in blue and red are part of the valine and isoleucine degradation pathways, respectively. Genes and Gene cluster dyed in black are part of the common degradation pathway. Gene cluster parts dyed in blue and red are designated to the valine and isoleucine degradation pathway. Assignment of genes to specific reactions was done using the Biocyc database and homology searches with blastp as well as on experiments from this study.

**FIGURE 5:**
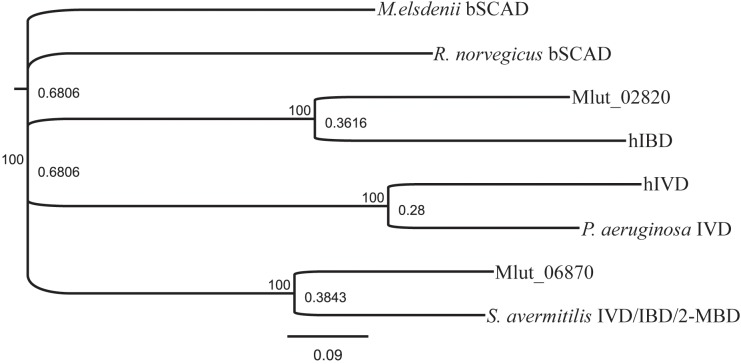
Similarity tree derived from amino sequence alignment of short-chain acyl-CoA- dehydrogenases. The sequences used are candidates found in the genome of *M. luteus* and selected, well-described examples from the literature. GenPept sequence record numbers are: *S. avermitilis* IVD/IBD/2-MBD, AAD44196; R. norvegicus bSCAD, AAA40669; Mlut_02820, ACS29839; hIBD, NP_055199; hIVD, 1IVH; *P. aeruginosa* IVD, NP_250705; Mlut_06870, ACS30219; *M. elsdenii* bSCAD, 1BUC. Alignment was performed using Clustal Omega with a Blosum62 matrix. Conversion in a phylogenetic tree was done in Geneious using bootstraping with 1000 replications.

In order to identify the genes encoding the enzymes of the downstream part of the BCAA degradation routes in *M. luteus*, we searched the genome for putative short-chain acyl-CoA dehydrogenases. We identified 7 candidate ORFs and inspected their genomic surroundings. Of the 7 candidate short-chain acyl-CoA dehydrogenases, 3 were found in gene clusters potentially involved in the degradation of BCAAs, i.e., ORFs Mlut_02820, Mlut_02900 and Mlut_06870. Mlut_02820 and Mlut_02900 were located near a set of genes which seem to encode almost all steps of the isoleucine/valine degradation pathways, and Mlut_06870 was found in a gene cluster which appears to encode all steps of the leucine degradation pathway (**Figure 4**). Amino acid sequence alignments of these dehydrogenases with homologs with known substrate specificities supported the annotation of Mlut_02820 as isobutyryl-CoA dehydrogenase (valine degradation), while Mlut_06870 was most similar to an enzyme from *Streptomyces avermitilis* with broad substrate specificity (Djordjevic et al., 1995; Tiffany et al., 1997; Zhang et al., 1999; Battaile et al., 2002; Battaile et al., 2004; Förster-Fromme and Jendrossek, 2008) (**Figure 5**).

Next, we generated deletion strains for all seven predicted short-chain acyl-CoA dehydrogenase ORFs by allelic exchange with a kanamycin marker. Some of these deletions were introduced also in a *M. luteus* trpE16 strain with elevated expression of the *ole* genes, named *M. luteus* ope (for olefin promoter exchange). The effect of each deletion on the isomeric composition of fatty acids and olefins in *M. luteus* allowed us to further specify the substrate specificities of the dehydrogenases. Additional experiments were performed, i.e., addition of BCAA to the growth medium and deletion of putative transcriptional regulators in the BCAA degradation gene clusters, in order to reveal the phenotypes of the generated mutants and to aid in deducing the substrate specificity of the acyl-CoA dehydrogenases. Due to their localization in putative BCAA degradation gene clusters, we focused on the deletion mutants of ORFs Mlut_02820, Mlut_02900 and Mlut_06870.

**FIGURE 6:**
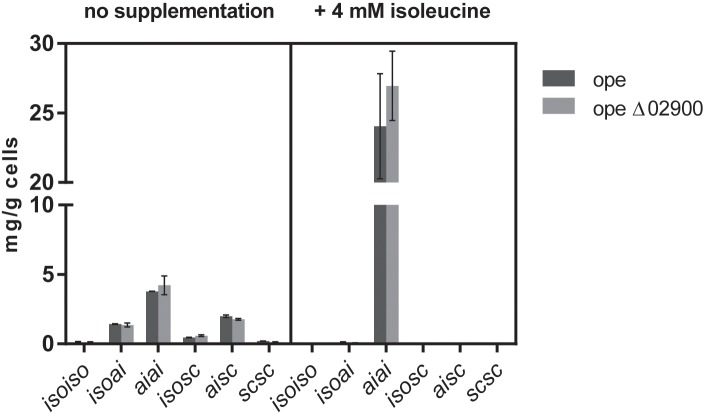
Distribution of fatty acid and olefin isomers in *M. luteus* mutant strains. **(A)** Fatty acids profile of selected mutants of the BCAA degradation pathways grown in minimal and complex medium. **(B)** Distribution of olefin isoforms in selected mutants of the BCAA degradation pathways grown in complex and in minimal medium without (–) and with (+) supplementation with 4 mM leucine, valine or isoleucine. Standard deviations did not exceed 4% except for ope in complex medium, which was less than 10%.

Under the growth conditions used by us, notable changes in the amounts and/or the isomeric composition of fatty acids and olefins could be observed only for the deletion mutant Δ06870:kan. Compared to the parental strain, the Mlut_06870 deletion mutant displayed a shift toward *iso*-branched odd-numbered fatty acids (from leucine-derived acyl-CoA primer) and in consequence a shift toward *isoiso*-branched olefins. These shifts in the fatty acids and olefin profiles may be explained by the increased supply of the leucine-derived acyl-CoA primer (isovaleryl-CoA) which is expected to accumulate if its further degradation is blocked by the deletion of the isovaleryl-CoA dehydrogenase. This was further supported by leucine supplementation experiment, where in the mutant the leucine-caused shift to *isoiso*-olefins was more pronounced (**Figure 6**).

Under our standard growth conditions, the deletions of Mlut_02900 and of Mlut_02820 did not have a measurable effect on the olefin or on the fatty acid profile of *M. luteus* (**Figure 6**). However, valine supplementation revealed marked differences in the branching pattern of the olefins of both Δ02900:kan and Δ02820:kan strains in comparison to the parental strain. In the parental strain, valine addition led to a shift to almost exclusively *isoiso*-olefins (and *iso*-even fatty acids), while in the Δ02900:kan and Δ02820:kan strains this shift was less pronounced and the mutants had a higher proportion of *isoai*- and *aiai*-olefins (**Figure 6B**). The observed olefin profiles of Δ02900:kan and of Δ02820:kan after valine supplementation are in agreement with a specificity of these dehydrogenases toward *ai*-acyl-CoA derivatives (2-methyl-butyryl-CoA, isoleucine degradation).

**FIGURE 7:**
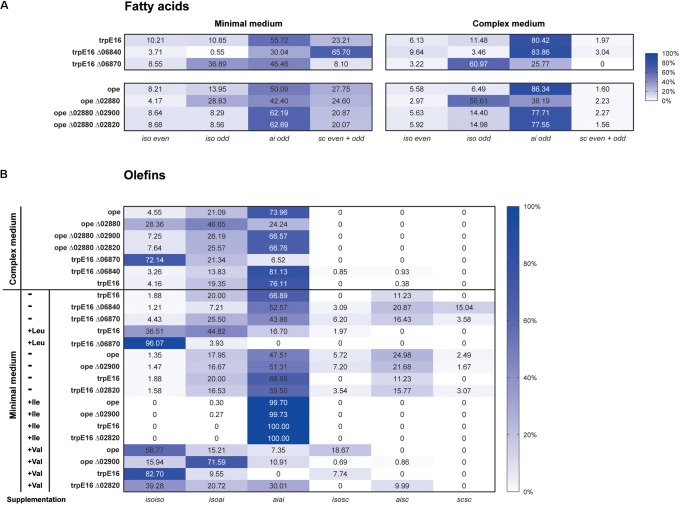
Olefin amounts of dehydrogenase mutant ope Δ02900:kan in comparison to the parental strain ope grown in minimal medium with and without supplementation of 4 mM isoleucine. Each bar represents the data of two biological replicates, error bars represent standard deviation.

Interestingly, supplementation of the minimal medium with 4 mM isoleucine resulted in both cases, the reference strain as well as in the Mlut_02900 deletion strain, in a threefold higher total olefin production (from ∼8 mg per gram dry cell weight without supplementation to ∼25 mg per gram cells with isoleucine supplementation) of almost exclusively the anteisoanteiso- (*aiai-*) configuration type (**Figure 7**), apparently due to the overall metabolic preference of *M. luteus* for isoleucine derivatives. Similar increase in olefin amounts could be observed also after supplementation with isoleucine of wild type and Mlut_02820 deletion mutant cultures.

Further evidence about the specificity of the acyl-CoA dehydrogenases of the Mlut_02820-2900 gene cluster was obtained by deleting the potential transcriptional regulator Mlut_02880 which acts as a repressor of all adjoining genes of this cluster. This regulation scheme could be revealed by qPCR and was confirmed by RNAseq data (unpublished results). A single deletion of the regulator (Δ02880:hyg) as well as double deletion mutants of the regulator and each of the two dehydrogenases of the cluster were generated in a *M. luteus* ope background and were further investigated by GC/MS. Compared with the control strain, deletion of the regulator gene Mlut_02880 led to an increase of leucine-derived (*iso*-branched odd-numbered) and to a drop of valine-derived (*iso*-branched even-numbered) and isoleucine-derived (*ai*-branched odd-numbered) fatty acids (**Figure 6A**). With respect to olefin production this resulted in an almost uniform distribution of *isoiso*- and *aiai*-branched olefins (**Figure 6B**). These changes in the isomer composition of the fatty acids and olefins can be explained by higher activities of acyl-CoA dehydrogenases with specificities toward the valine- and isoleucine-derived acyl-CoAs in the regulator deletion strain. In agreement with this interpretation, the additional deletion of either one of the two nearby dehydrogenase genes (Mlut_02900, Mlut_02820) in the Δ02880:hyg background fully restored the fatty acid/olefin isomer profile of the wild-type.

### Growth Studies With BCAA as Nitrogen Source

We hypothesized that, if an externally supplied BCAA is the only source of nitrogen, the ability of *M. luteus* to grow in such medium would be indicative of its ability to catabolize the supplied amino acid (Dawes, 1952). The *M. luteus* strain used by us was unable to grow in glucose-containing medium with isoleucine or valine as the only source of nitrogen, while good growth could be observed with leucine. We assumed that the lack of growth on isoleucine and valine may be due to the continuous repression of the degradation gene cluster Mlut_02820-2900 by the regulator Mlut_02880. However, an additional growth study using the regulator deletion strain revealed that also under conditions of induction of the isoleucine and valine degradation cluster growth was still not possible on these amino acids (data not shown).

**FIGURE 8:**
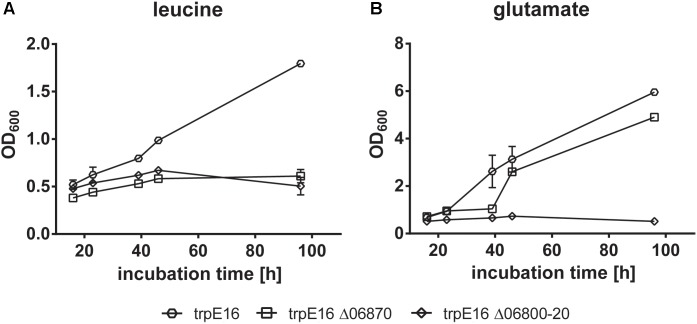
Growth curves of BCKD (Mlut_06800-20) and isovaleryl-CoA dehydrogenase (Mlut_06870) deletion mutants in glucose minimal medium supplemented with leucine **(A)** or glutamate **(B)** as the only nitrogen source. Each curve represents data from two biological replicates, error bars represent standard deviation.

Growth with leucine was no longer possible for the Mlut_06800-06820 and for the Mlut_06870 deletion mutants. Deletion of the BCKD complex (Mlut_06800-06820) on one hand prevents the formation of branched-chain fatty acid primer molecules from any supplemented amino acid and on the other hand leads to the accumulation of metabolic intermediates (α-keto acids, substrates of the BCKD reaction) which presumably inhibits further deamination of amino acids and formation of glutamate, which is crucial for the biosynthesis of other amino acids. Therefore, growth of this mutant in minimal medium with supplementation of any of the tested amino acids did not occur. Interestingly, glutamate supplementation also did not allow growth of the BCKD mutant (**Figure 8**). As shown previously (see above), deletion of Mlut_06870 did not prevent free fatty acid synthesis, but this block in leucine catabolism may lead to the accumulation of the upstream intermediates and thereby inhibit a sufficient (i.e., growth-supporting) extent of deamination of leucine and formation of the central N-metabolite glutamate. In accordance, the supplementation with glutamate allowed growth of *M. luteus* trpE16 Δ06870:kan.

### Mlut_06840 Is a Possible Repressor of Cluster Mlut_06850-06890

The ORF Mlut_06840 codes for a putative transcriptional regulator and is located near the predicted leucine degradation genes Mlut_06850-06890 and simultaneously in the vicinity of the BCKD complex-encoding genes Mlut_06800-06820 (see **Figure 4**). This putative regulator belongs to the TetR family whose members are known to control catabolic pathways.

**FIGURE 9:**
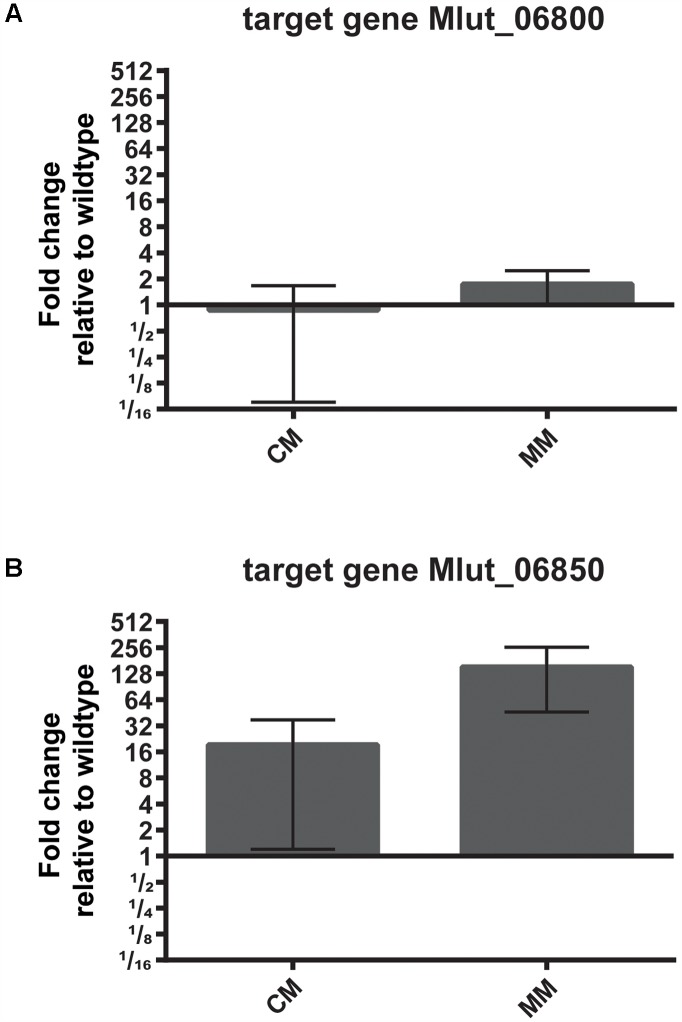
Effect of deletion of the transcriptional regulator Mlut_06840 on the expression levels of Mlut_06800 **(A)** and Mlut_06850 **(B)**. Gene expression was measured by qPCR and is expressed relative to the parental strain, using the 2^-ΔΔ*C*_T_^ method. The mean of three technical replicates from two biological samples is shown, error bars are standard deviation. A log_2_-scale was employed on the *y*-axis. The strains were grown in complex medium (CM) or minimal medium (MM).

To identify possible targets of the regulator encoded by Mlut_06840 among the surrounding branched amino acid degradation genes, the levels of expression of Mlut_06800 and Mlut_06850 in the deletion mutant trpE16 Δ06840 (markerless) and in the parental strain trpE16 were analyzed by qPCR. Relative quantification by the 2^-ΔΔ*C*_T_^ method revealed that the product of Mlut_06840 acts as a repressor on the genes of the cluster Mlut_06850-90, but not on Mlut_06800-06820, since deletion of Mlut_06840 led to an induction of the leucine degradative pathway (**Figure 9**). On the level of fatty acids, the induction of the Mlut_06850-06890 gene cluster during growth in minimal medium led to a shift from branched fatty acid production, which derive from any kind of BCAA, to *sc* fatty acid production. In complex medium the deletion of Mlut_06840 negatively affected only the odd-numbered leucine-derived fatty acids (**Figure 6A**). Also, regarding olefin production, the induction of Mlut_06850-06890 in the Mlut_06840 mutant led to less branched-chain and to more straight chain olefin isomers in minimal medium, which reflects the shift from branched to more straight chain fatty acid production (**Figure 6B**).

## Discussion

A characteristic feature of the olefins produced by *M. luteus* is their terminal *iso-* or *ai*-branching. Using mutation of selected genes/operons of amino acid catabolism identified by *in silico* genome sequence analysis, and cellular composition analysis, this work demonstrates the importance of the branched amino acid catabolism in *M. luteus* for the branching pattern of these potentially valuable hydrocarbons. This work represents the first detailed study about the genetic basis of branched-chain fatty acid synthesis and BCAA metabolism in a member of the phylum *Actinobacteria*.

One key player is the BCKD complex, which generates the branched acyl-CoA primer molecules needed for fatty acid as well as for polyketide biosynthesis. It has been shown previously that the BCKD reaction can be used to modify the pattern of heterologously expressed, fatty acid-derived hydrocarbons in *E. coli* and also of naturally produced polyketides (Denoya et al., 1995; Howard et al., 2013). Therefore it was reasonable to evaluate the influence of this reaction also on the fatty acid-derived olefin synthesis in *M. luteus*.

By homology searches, two potential candidates were found, one encoded by gene cluster Mlut_06800-06820, and the other by Mlut_17810-17790. Promoter-up mutants displayed elevated gene expression of the clusters, but only in the case of Mlut_06800-06820 this led to the alleviation of the olefin synthesis limitation in minimal medium, allowing an increase of olefin production by 200% (**Figure 1B**). A limitation for olefin production thus apparently comes from a lack of primer molecules for fatty acid biosynthesis, which is relieved by enhancing the expression of the BCKD complex. This reveals Mlut_06800-20 as the correct BCKD complex-encoding cluster among the possible candidate gene clusters on the *M. luteus* chromosome. Since the elevated expression of the BCKD gene cluster had no significant effect on the branching isomer composition of fatty acids and olefins this enzyme complex does not appear to have substrate preferences for specific amino acid derivatives.

Further experimental evidence in support of the annotation of the cluster Mlut_06800-06820 as the BCKD complex is provided by our BCAA supplementation experiments. In the Mlut_06800-06820 deletion mutant the synthesis of branched fatty acids as well as olefin synthesis was strongly inhibited, most likely due to the missing supply of branched acyl-CoA primer molecules for fatty acid synthesis. Supplementation of the medium with one of the three branched amino acids led to the corresponding changes of the fatty acid branching pattern only in the parental strain but not in the Mlut_06800-06820 deletion mutant (**Figure 3A**). Residual production of branched-chain fatty acids could be observed in this mutant, presumably as a result of unspecific activity of the pyruvate-/2-oxoglutarate dehydrogenase complex. The reduced fatty acid synthesis and simultaneous shift to a higher proportion of *sc* fatty acids in the absence of the BCKD complex indicates that acetyl-CoA served as the major acyl-CoA primer for fatty acid biosynthesis and may reflect the fact that FabH (β-ketoacyl-ACP synthase III) in *M. luteus* favors branched acyl-CoA primers as substrate (Pereira et al., 2012).

Using the BCKD deletion mutant, we could show that in *M. luteus* an alternative route leading to branched-chain fatty acid primers is present. This pathway probably utilizes short- and branched-chain-specific acyl-CoA synthetases. The short branched-chain carboxylic acids 2-methylbutyric acid, isobutyric acid and isovaleric acid added to the medium were apparently directly converted to the corresponding acyl-CoA primer molecules, enabling the synthesis of the corresponding branched fatty acids and olefins in the BCKD deletion mutant (**Figure 3B**).

Other enzymes of importance for the fate of branched acyl-CoA primer molecules for fatty acid biosynthesis are the short- and branched-chain-specific acyl-CoA dehydrogenases. These catabolic enzymes compete for the same substrates also used as primers in branched fatty acid biosynthesis. Among the putative acyl-CoA dehydrogenases encoded in the genome of *M. luteus*, those derived from the ORFs Mlut_02820 and Mlut_06870 possessed short-chain specific domains.

The acyl-CoA dehydrogenase Mlut_06870 is part of a gene cluster which contains genes for most of the activities needed for leucine degradation (**Figure 4**). The gene product exhibits a higher level of amino acid sequence similarity with the dehydrogenase from *S. avermitilis*, which has been shown to have a broad specificity for various short branched acyl-CoA substrates (Zhang et al., 1999), than with the well characterized human isovaleryl-CoA-dehydrogenase (Tiffany et al., 1997). The growth studies with supplemented amino acids as the only nitrogen source (**Figure 8**) demonstrated a growth defect of the Mlut_06870 deletion mutant on leucine. The deletion of Mlut_06870 and the presumably resulting elevated concentration of isovaleryl-CoA led to a greater share of *iso*-branched odd-numbered fatty acids, which are leucine derivatives, and in consequence to a higher share of the *isoiso*-configuration type of the olefins. The specificity of the Mlut_06870-encoded dehydrogenase for isovaleryl-CoA could further be confirmed at the olefin level by supplementation with leucine, which led to a higher proportion of *isoiso*-olefins in the deletion strain in comparison to the parental strain. Based on these results, we conclude that the dehydrogenase encoded by Mlut_06870 is specific for the leucine degradation acyl-CoA derivative (isovaleryl-CoA) and is an important component in the *ai*-dominated fatty acid and olefin pattern of *M. luteus*.

The regulator Mlut_06840 was found to be a repressor of the leucine degradation gene cluster Mlut_06850-06890, and its deletion obviously resulted in minimal medium not only in a drop of leucine-, but also of isoleucine- and valine-derived metabolites in fatty acid and olefin production. This implies that the regulation brought about by the gene product of Mlut_06840 is not limited to gene cluster Mlut_06850-06890. In complex medium the lack of the Mlut_06840 ORF led to the induction of leucine degradation accompanied by a lower abundance of *iso*- fatty acids, which allowed a higher throughput of isoleucine-derivatives and an increase in aiai-olefin production. Despite the gain of knowledge about the regulator encoded by Mlut_06840, it presently cannot be used as a regulating screw to favorably manipulate branched fatty acid primer supply, because of additional unknown targets.

Mlut_02820 and Mlut_02900 are putative dehydrogenase ORFs found in a gene cluster encoding almost all activities required for valine and isoleucine degradation. Due to the high level of amino acid sequence similarity of Mlut_02820 to the well characterized human isobutyryl-CoA-dehydrogenase (Battaile et al., 2004) (see **Figure 5**) we reasoned that the encoded protein may be specific for valine metabolism. However, our experiments support the hypothesis that Mlut_02820 and Mlut_02900 dehydrogenases have activities with both the valine- and isoleucine-derived acyl-CoA derivatives. The dual specificity of these dehydrogenases is supported by the following observations: (i) deletion of each one had no impact on the fatty acid or on the olefin production pattern and these deletion mutants showed the same phenotype in the amino acid supplementation experiments, (ii) the deletion of the transcriptional regulator Mlut_02880, for which we have evidence acts as a repressor of all the genes in the cluster, led to a decrease in both *iso*-even (valine derivatives) and *ai*-odd (isoleucine derivatives) fatty acids, and (iii) introducing the single deletions of Mlut_02820 and Mlut_02900 in the Mlut_02880 deletion strain restored in both cases the fatty acids profile of the wild type. However, because our conclusions are based mainly on fatty acids profile analysis of mutants, the precise function assignment for Mlut_02820 and Mlut_02900 has to be further clarified by enzyme purification and detailed biochemical characterization.

In summary, we could identify and functionally annotate the *M. luteus* genomic regions containing the genes of the BCKD complex, leucine degradation, and isoleucine/valine degradation, which play key roles in the branched amino acid catabolism in this organism. In addition, the influence of these catabolic routes on the fatty acid and olefin synthesis could be pointed out. Importantly, we found conditions leading to substantially increased olefin production in *M. luteus* and demonstrated ways to manipulate the isomeric composition of these molecules. This work can serve as a basis for further improvements in the production of customized biofuels in bacteria.

## Author Contributions

MS and AA contributed to the design of the work and to the acquisition, analysis, and interpretation of data. They revised the work critically for important intellectual content. They agreed to be accountable for the work in points of accuracy and integrity. They gave their approval for the final version to be published. PS contributed to the acquisition, analysis, and interpretation of data. He revised the work critically for important intellectual content. He agreed to be accountable for the work in points of accuracy and integrity. MÜ contributed to the acquisition and analysis of data. She agreed to be accountable for the work in points of accuracy and integrity. WL contributed to the design of the work and to the interpretation of data. He revised the work critically for important intellectual content. He agreed to be accountable for the work in points of accuracy and integrity. He gave his approval for the final version to be published.

## Conflict of Interest Statement

The authors declare that the research was conducted in the absence of any commercial or financial relationships that could be construed as a potential conflict of interest.
